# An unusual cause of gastric outlet obstruction: A pancreaticoduodenal artery aneurysm

**DOI:** 10.4103/0256-4947.55171

**Published:** 2009

**Authors:** Abdulaziz Alhasan, Patan M. Khan

**Affiliations:** a From the Department of Internal Medicine, Faculty of Medicine, Umm Al-Qura University, Makkah, Saudi Arabia; b From the Department of Internal Medicine, King Fahad Armed Forces Hospital, Jeddah, Saudi Arabia

## Abstract

We present a rare case of gastric outlet obstruction due to compression of the duodenum by a pancreaticoduodenal artery (PDA) aneurysm 2.5 cm in diameter, in a 43-year-old man from Saudi Arabia who presented with persistent vomiting and epigastric pain. The initial investigations and blood works were negative, and esophagogastroduodenoscopy (EGD) was unremarkable. A CT abdomen demonstrated a mass around the duodenum and dilatation of the stomach, and CT angiography showed the PDA aneurysm. The patient was stabilized and then referred to a tertiary center for embolization. Our case demonstrates a diagnostic challenge that physicians may encounter in patients who present with vomiting and epigastric pain.

Pancreaticoduodenal artery (PDA) aneurysm is a rare type of visceral artery aneurysm, which also includes splenic, renal, hepatic, mesenteric, and aortic arteries aneurysms.^1^ PDA aneurysms have nonspecific clinical presentations,^2^ which explains why they are not usually considered in the differential diagnosis of patients who present with epigastric pain or vomiting. We report a case of a patient with a PDA aneurysm who presented with repeated vomiting and epigastric discomfort. The aneurysm was found incidentally in a CT scan of the abdomen. Visceral angiography was done to visualize the aneurysm and the diagnosis of PDA aneurysm causing a gastric outlet obstruction was confirmed. The patient was referred to a specialized center and was successfully treated.

Although the PDA aneurysm was reported in many cases, the presentation as gastric outlet obstruction is rare. Commonly, gastric outlet obstruction is caused by gastric and peripancreatic malignancies in developed and developing countries.^3^ Peptic ulcer disease is the most common benign cause of gastric outlet obstruction.^4^ We present this case to draw attention to the fact that PDA and other visceral aneurysms are rare causes of gastric outlet obstruction, which should be considered if none of the more common causes of gastric outlet obstruction can be identified.

## CASE

A 48-year-old Saudi man presented with repeated vomiting and epigastric discomfort for a duration of ten days. Vomiting occurred one hour after eating, and was projectile, yellowish in color, and contained undigested food with no mucus or blood. It was associated with mild epigastric discomfort, which was of gradual onset, intermittent, dull and aching in nature, and occasionally felt in the back and both hypochondria. There was no history of peptic ulcer disease or drug ingestion. The patient denied any history of fever, cough, weight loss, or trauma. He was diagnosed with hypertension 10 months before presentation for which he was taking fosinopril 10 mg once daily. The family history was negative for aneurysms and malignancies. The patient was retired from the military services. He drank alcohol occasionally and had a 10 pack-year smoking history.

**Figure 1:**
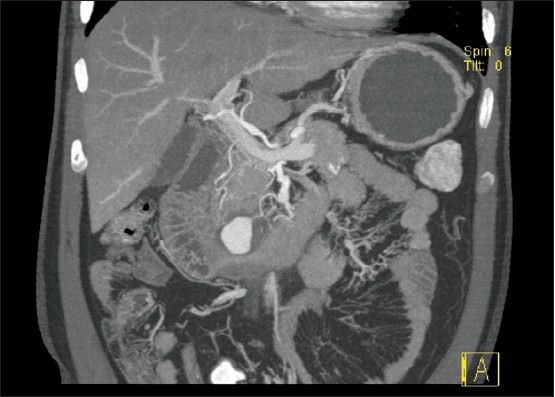
CT of the abdomen showing the PDA aneurysm.

**Figure 2:**
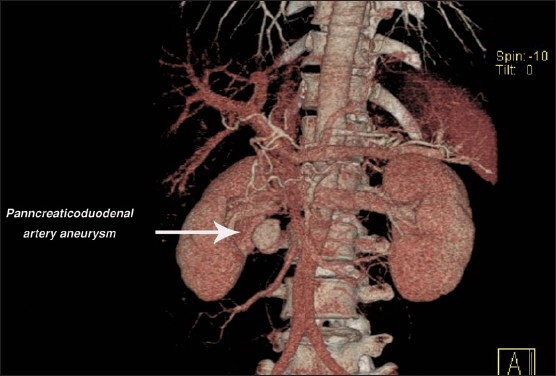
CT angiography slowing the pancreaticoduodenal artery aneurysm.

Clinical examination revealed a middle-aged man who was fully conscious, afebrile, and hemodynamically stable. Systemic examination revealed no abnormality, but abdominal examination revealed mild tenderness in the epigastric area. The rectal examination was unremarkable. Blood tests were unremarkable (Table 1) Esophagogastroduodenoscopy revealed hiatus hernia with mild gastritis not consistent with the clinical condition of the patient. A gastric biopsy was negative for malignancy. A CT scan of the abdomen demonstrated a rounded structure in the posterior aspect of the pancreas, anterior to the third part of duodenum, which measured 2.5 cm and compressed the duodenum, causing dilatation in the stomach, and the first and second part of the duodenum, which suggested an aneurysm in PDA or gastroduodenal (hepatic) artery (Figure 1). A CT angiography suggested by the radiologist confirmed the diagnosis of PDA aneurysm causing gastric outlet obstruction (Figure 2). The patient received supportive treatment and was referred to a tertiary center for embolization of the aneurysm.

**Table 1:** Laboratory investigations.

Complete blood count and coagulation profile
White blood cell count: 17.8×10^3^ cells per mm^3^
Hemoglobin: 11.7 g/dL
Hematocrit: 35.2%
Platelets: 585 000 per mm^3^
Mean corpuscular volume: 65.8 fL
Mean corpuscular hemoglobin: 21.9 pg
Prothrombin time: 14.6 seconds
International normalized ratio: 1.09
Partial thromboplastin time: 34.4 seconds
**Renal profile**
Sodium: 142 meq/L
Potassium: 3.7 meq/L
Chloride: 100 meq/L
Bicarbonate: 27 meq/L
Urea: 6.8 mmol/L
Creatinine: 89 μmol/L
Glucose: 7.2 mmol/L
Anion gap: 15
**Liver function tests**
Lactate dehydrogenase: 404 U/L
Alanine aminotransferase: 107 U/L
Alkaline phosphatase: 163 U/L
Albumin: 44 g/L
Total protein: 80 g/L (60-85 g/L)
Amylase: 77 U/L

## DISCUSSION

PDA aneurysms are very rare. They comprise 2% of all visceral artery aneurysms.^5^ Since Ferguson et al reported the first case of PDA aneurysm in 1895,^6^ 88 cases of PDA aneurysms were reported in the English literature until 1993 and 52 cases of PDA aneurysms were reported between 1973 and 1999.^7^ More reported cases increased awareness as to the importance of early detection before rupture.

Men are four times as likely as women to have PDA aneurysms.^8^ The vast majority of patients with these aneurysms experience epigastric pain and discomfort. This may be secondary to underlying pancreatic disease in 30% of PDA aneurysms.^8^ Other clinical presentations include hemosuccus pancreaticus–which is the presence of bleeding into the pancreatic duct–or hemobilia, jaundice, and shock.^9^

Reported cases of PDA aneurysms have also included a case that presented with vaginal bleeding and incidentally was found to have a pulsatile mass on ultrasound examination.^10^ Another case presented with an incarcerated inguinal hernia that subsequently hemorrhaged into the retroperitoneum from a ruptured PDA aneurysm.^10^ Itoh et al reported a case of PDA aneurysm causing pancreatic pseudotumor and duodenal obstruction, which on angiography was an aneurysm of 8 mm in diameter, found in the posterior superior PDA.^11^ Androulakakis et al reported a case of a gastric outlet obstruction caused by giant gastroduodenal artery aneurysm.^12^ Chiou et al reported a case of pancreaticoduodenal artery aneurysm that began with intestinal angina and weight loss.^13^

The most common cause of these aneurysms is pancreatitis-related vascular necrosis or vessel erosion by an adjacent pancreatic pseudocyst.^8^ Othe causes also include atherosclerosis, infection, congenital defects, fibromuscular dysplasia, connective tissue disorders (polyarteritis nodosa or Takayasu arteritis) and trauma.^1^,^8^ Our patient had atherosclerosis risk factors which included a past history of hypertension and a 10-pack year smoking history. Alcohol consumption is also a risk factor for pancreatitis; Any of these factors might have been the cause of his aneurysm.

**Figure 3:**
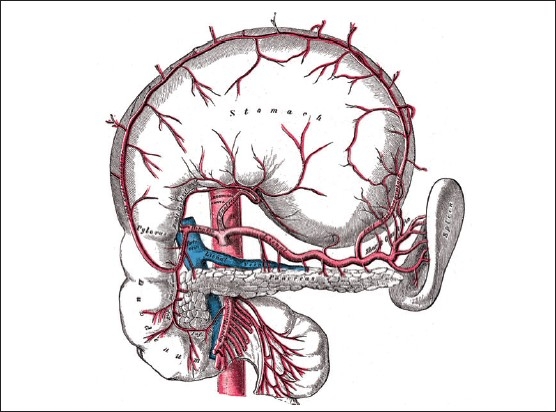
The superior and inferior PDA arteries pass close to the duodenum and gastric outlet.

Gastric outlet obstruction is commonly caused by gastric malignancy or stenosis of the pylorus as a complication of chronic peptic ulceration.^14^ Other benign differential diagnoses of gastric outlet obstruction include infections such as tuberculosis and infiltrative diseases such as amyloidosis. Gastric outlet obstruction may also be caused by gallstone, a condition termed as Bouveret syndrome. Superior and inferior PDAs communicate anteriorly to the head of the pancreas and medial to the first part of the duodenum (Figure 3).^15^ Arteriography is necessary to confirm the existence of PDA aneurysms. CT or magnetic resonance angiography (MRA) are also of importance in recognizing these aneurysms and are helpful in detecting the presence of rupture or associated pancreatic disease.^10^ In our case, a contrast-enhanced CT scan of the abdomen revealed the aneurysm from the PDA, which has a blood supply from both the superior mesenteric artery and gastroduodenal artery and measures 2.5 cm, causing narrowing of the transverse duodenum with dilatation of stomach, and the first and second part of the duodenum.

We believe that PDA aneurysms and other visceral aneurysms should be considered in any patient who presents with repeated vomiting and gastric outlet obstruction symptoms, especially if no cause can be found.
